# Buprenorphine/naloxone access for people with opioid use disorder in correctional facilities: taking steps to support knowledge translation

**DOI:** 10.1186/s40352-022-00174-w

**Published:** 2022-03-02

**Authors:** Lori Regenstreif, Marina Sadik, Erin Beaulieu, Claire Bodkin, Lori Kiefer, Dale Guenter, Patsy W. P. Lee, Fiona G. Kouyoumdjian

**Affiliations:** 1 Department of Family Medicine, McMaster University, 100 Main Street West, 5th Floor, Hamilton, ON L8P 1H6 Canada; 2 Dalla Lana School of Public Health, University of Toronto, Toronto, Ontario Canada; 3 Ontario Ministry of the Solicitor General, Toronto, Ontario Canada; 4 Michael G. DeGroote School of Medicine, McMaster University, Hamilton, Ontario Canada

**Keywords:** Opioid use disorder, Opioid agonist treatment, Buprenorphine, Correctional facility, Prison, Jail, Addiction treatment

## Abstract

People with opioid use disorders are overrepresented in correctional facilities, and are at high risk of opioid overdose. Despite the fact that buprenorphine/naloxone is the first line treatment for people with opioid use disorder, there are often institutional, clinical, and logistical barriers to buprenorphine/naloxone initiation in correctional facilities. Guided by the knowledge-to-action framework, this knowledge translation project focused on synthesizing knowledge and developing a tool for buprenorphine/naloxone initiation that was tailored to correctional facilities, including jails. This information and tool can be used to support buprenorphine/naloxone access for people in correctional facilities, in parallel with other efforts to address barriers to treatment initiation in correctional facilities.

## Introduction

Opioid use disorder, a chronic, complex condition in which there is uncontrolled use of opioids despite harmful consequences (American Psychiatric Association, 2022), is common in people in correctional facilities. Opioid agonist treatment in this population, for example methadone or buprenorphine/naloxone, has been shown to reduce the risk of adverse outcomes, including mortality, non-fatal overdose, HIV infection, illicit drug use, and criminal activity (Garcia et al., 2007; Gordon et al., 2014; Green et al., 2018; Hedrich et al., 2012; Magura et al., 2009; Marsden et al., 2017; Sullivan et al., 2008; Zaller et al., 2013). Even in the current context of the dominance of high-potency synthetic fentanyl analogues in the North American illicit drug market, evidence indicates that opioid agonist treatment protects against overdose toxicity deaths (Pearce et al., 2020).

In Canada, national guidelines recommend buprenorphine/naloxone as first line pharmacological treatment for opioid use disorder, based on treatment effectiveness and safety (Bruneau et al., 2018). However, buprenorphine/naloxone treatment access and quality are frequently suboptimal in correctional facilities in the USA and Canada for multiple reasons (Correctional Service of Canada,^ 2019^; Nunn et al., 2009; Ontario Ministry of the Solicitor General, 2019). There may be challenges related to short or uncertain lengths of stay, for example for people who are remanded with an unknown date of release. There may be institutional barriers related to prescribing policies and health care accessibility, and inadequate processes and relationships to support continuity of care at the time of release. There may be clinical issues such as health care provider knowledge and values, patient knowledge and values, and medical challenges such as recent polysubstance use and intoxication (Kouyoumdjian et al., 2018; Nunn et al., 2009). Finally, there may be logistical challenges to providing access to buprenorphine/naloxone initiation at the time of admission, when opioid withdrawal symptoms may be most prominent.

**Fig. 1:**
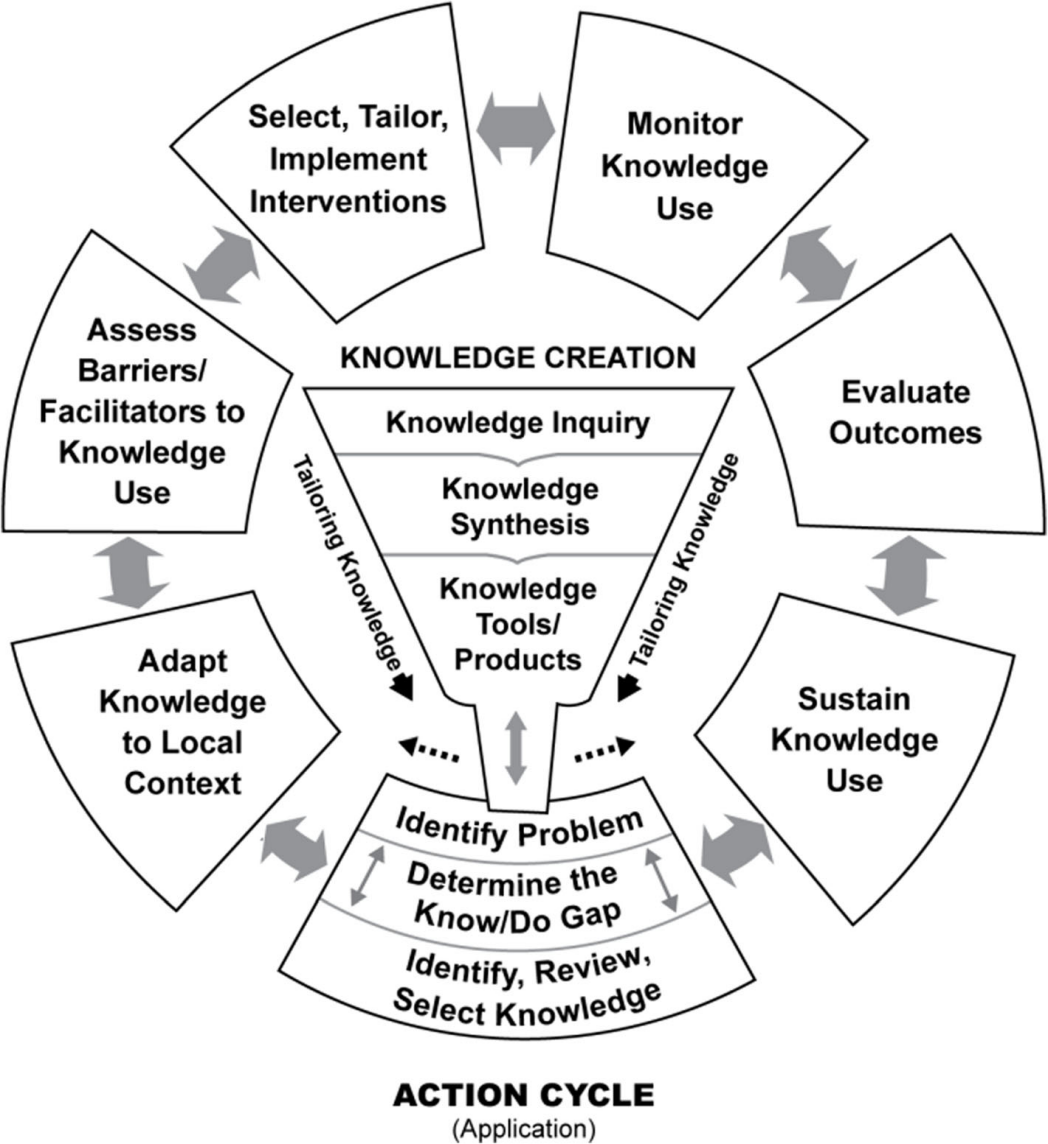
The Knowledge-to-action cycle. Reprinted from Straus SE, Tetroe J, Graham I. Knowledge translation in health care: moving from evidence to practice. 2nd ed. BMJ Books, Wiley, 2013. Reprinted with permission. Copyright© 2013, John Wiley and Sons

While various strategies are indicated to improve buprenorphine/naloxone access in correctional facilities, the knowledge-to-action cycle (Fig. 1) provides a framework to address barriers specifically related to knowledge implementation (Straus et al., 2013). In this knowledge translation project, we moved iteratively between knowledge creation and the action cycle. Knowledge creation, shown in the funnel, refers to the process of refining and summarizing information tailored to the needs of the end user. The action cycle, shown in the outside ring, includes the activities needed for knowledge implementation. In this project we identified a specific knowledge gap related to the initiation of buprenorphine/naloxone in correctional facilities, and then we synthesized relevant literature, adapted knowledge and guidance on buprenorphine/naloxone initiation to the local context of correctional facilities including jails, and developed a knowledge tool.

## Work on phases of the knowledge-to-action cycle

### Action cycle phase: identify problem

Clinical guidelines for buprenorphine/naloxone initiation focus on people who are in withdrawal, recommending an initial treatment dose and a rate of dose titration based upon withdrawal scores (Julie Bruneau et al., 2018; J. Bruneau et al., 2018; Center for Substance Abuse Treatment, 2004; Kampman & Jarvis, 2015), i.e.*,* based on the Clinical Opiate Withdrawal Scale (COWS). For patients in correctional facilities in a state of acute withdrawal from opioids, this guidance applies well.

However, guidelines do not cover a clinical scenario that occurs frequently in correctional facilities: the initiation of buprenorphine/naloxone treatment for people who have lost their physical dependence on opioids. People who routinely use opioids lose their physical dependence and respiratory tolerance after only a few days without ongoing exposure to opioids, increasing their susceptibility to the respiratory depressant effects of a subsequent exposure. On admission to correctional facilities, people eligible for buprenorphine/naloxone treatment may not be offered treatment or they may decline treatment, and the provider and patient may decide to initiate treatment only after withdrawal symptoms have abated and physical dependence and tolerance are reduced or absent.

### Knowledge creation phase: knowledge synthesis

To address this information gap, we reviewed and summarized published research describing protocols for buprenorphine/naloxone treatment initiation and titration in people with opioid use disorder who have lost their physical dependence and tolerance. We conducted a focused literature search and contacted authors of some identified articles. We identified four studies (Table 1), all of which were conducted in the USA.

**Table 1:** Research evidence on buprenorphine/naloxone initiation for people in correctional facilities with OUD who were not opioid dependent or tolerant

Study	Study type	Participants	Treatment procedures	Efficacy
Garcia et al., 2007	Intervention with no control group: daily bup/nal in prison and post-release	42 males in total, most were opioid tolerant, with ~ 6 months prior to release	Treatment was initiated pre-release. Opioid nontolerant participants were initiated at 2 mg, and increased by 2 mg increments prn. They were assessed 1x/week in first month, then every 2 weeks until release. Goals were to reach a therapeutic dose and eliminate cravings.	Results were not stratified by opioid tolerance. Comparing drug use in the 30 days before incarceration and the 30 days after release, those who remained on treatment (*n* = 33) reported a median reduction in days of drug use of 22 for heroin use and 25 for cocaine use, whereas those who did not remain in treatment (*n* = 9) reported no reduction in days of heroin or cocaine use after release.
Springer et al., 2010	Intervention with no control group (nested in an RCT) comparing outcomes pre- and post-bup/nal treatment	People with HIV and OUD: 23 received bup/nal: 18 males and 5 females	Treatment was initiated around the day of release. Initial dose was 2 mg and dose was increased by 2 mg prn. Participants were assessed daily during induction and monthly thereafter.	91% of participants completed induction and 74% completed 12-week treatment. Likert scale rating of opioid craving reduced from mean 6.0/10 to 2.2/10 and satisfaction level was 9.5/10 throughout the 12 weeks. For those who completed induction: no change in undetectable viral load for those in treatment at 12 weeks compared to baseline: 61% vs. 63% log_10_ copies/mL, or negative urine drug screen: 83% vs. 71%.
Zaller et al., 2013	Intervention with two non-randomized groups: pre- and post-release initiation of treatment	44 people: 37 males and 7 females	12 subjects started treatment within 2 weeks pre-release and 32 subjects started treatment 3 days post-release. Dose was adjusted based on symptom review.	For those who initiated treatment in prison vs. post-release, mean time to first post-release prescriber appointment was 3.9 vs. 8.8 days. At the study conclusion 6 months after release, 91% of those who initiated treatment in prison remained in treatment vs. 34% of those who initiated treatmented post-release, and median post-release treatment duration was 24 vs. 9 weeks.
Vocci et al., 2015 Gordon et al., 2014	RCTof in-prison buprenorphine treatment vs. counselling only (series of 12 weekly sessions)	211 people with heroin dependence prior to imprisonment: 148 males and 63 females	Treatment was initiated 3–9 months pre-release. The standard protocol was induction at 1 mg, then dose increased 1 mg/week to 4 mg, then 2 mg/week to 8 mg, then to 16 mg after 2 weeks and administered every other day. If tolerated, dosing was changed to 3x/week. Dosing was changed based on side effects and patient requests.	Results were not stratified for those who were not opioid tolerant. Compared with those randomized to counseling only, those randomized to the buprenorphine treatment group were more likely to initiate treatment in prison: 99.0% started buprenorphine vs. 80.4% started counselling, and to continue treatment in the community after release: 47.5% vs. 33.7%. There was no difference in completing treatment in prison: 61.5% of the buprenorphine group remained in treatment at release vs. 63.6% of those randomized to counselling only.

We used the term tolerant or dependent based on the language used by the study authors. When dose is indicated as ‘Xmg’, this represents Xmg *buprenorphine* in a buprenorphine/naloxone ratio of 1/0.25

We determined that an appropriate starting dose for patients who have lost their opioid dependence and tolerance is between 2 mg and 4 mg; this is based on available evidence, the pharmacology of buprenorphine (as a partial agonist with a “ceiling effect” on respiratory depression (Center for Substance Abuse Treatment, 2004)), our clinical experience, and consultations with colleagues and people with lived experience of incarceration and opioid use disorder. Informed consent should be obtained, and should include discussion of possible side effects of the medication such as nausea, sedation, and euphoria. Patients should have their dose reassessed every few days and at least once per week.

### Action cycle phase: adapt knowledge to local context

Given the specific circumstances of the correctional facility and patient population, it is important to adapt knowledge and guidelines to enhance applicability and useability within this context.

In the community or hospital setting, reassessment of patients in withdrawal typically occurs within a few hours of treatment initiation. Recognizing the constraints of health care in many correctional facilities, we recommend reassessment within a wider time window of 2 to 12 h. Depending on what is feasible within that time window, the reassessment may focus on precipitated withdrawal symptoms, residual withdrawal symptoms, and positive response.

In correctional facilities, decisions regarding dose should take into account symptoms, patient preference, anticipated release date, and institutional and health care resources, aiming for a maintenance dose of at least 8 mg per day (Greenwald et al., 2014). Dosing should be titrated based on subjective symptoms of cravings or reported desire to use illicit opioids. For patients who have received buprenorphine in the past, previous doses at which they were clinically stable can inform the dosing strategy. A patient’s anticipated release date may also inform decisions regarding an initial dose and dose changes; patients and providers may feel some urgency to increase the dose quickly so that the patient reaches a therapeutic dose before release to the community.

Buprenorphine/naloxone initiation in correctional facilities must also explicitly include planning to support continuity of care at the time of release from correctional facilities. All patients who initiate buprenorphine/naloxone in correctional facilities will need a plan for access to treatment and health care on release. Recognizing the many competing priorities and other barriers to accessing care on release, correctional health care staff should facilitate relevant referrals to community-based health care to support timely treatment access and liaise with community health care providers. If feasible and acceptable in the context of local regulations and practice structures, correctional health care providers may provide bridging prescriptions or take-home doses for patients at the time of release until patients can access follow up. For any bloodwork ordered, we suggest sending copies to community physicians who are involved in the patient’s care, so that if the patient is released prior to receiving their test results, they will still have access to this important information. This will help facilitate any indicated treatment or follow up and prevent redundant testing, use of unnecessary resources, extra costs, and inconvenience. In addition, any test results that require follow up or follow up plans made prior to release could be provided directly to the patient to further support continuity of care.

We also note the need to offer counselling regarding safer drug use for all people who use drugs, including those with and without opioid use disorder and those who do and do not access opioid agonist treatment, and supports for access to harm reduction tools both in custody and in the community, including naloxone training and distribution.

### Action cycle phase: select, tailor, implement interventions

To support buprenorphine/ naloxone initiation in correctional facilities, we developed a tool that summarizes key considerations and evidence-informed best practices.

**Fig. 2:**
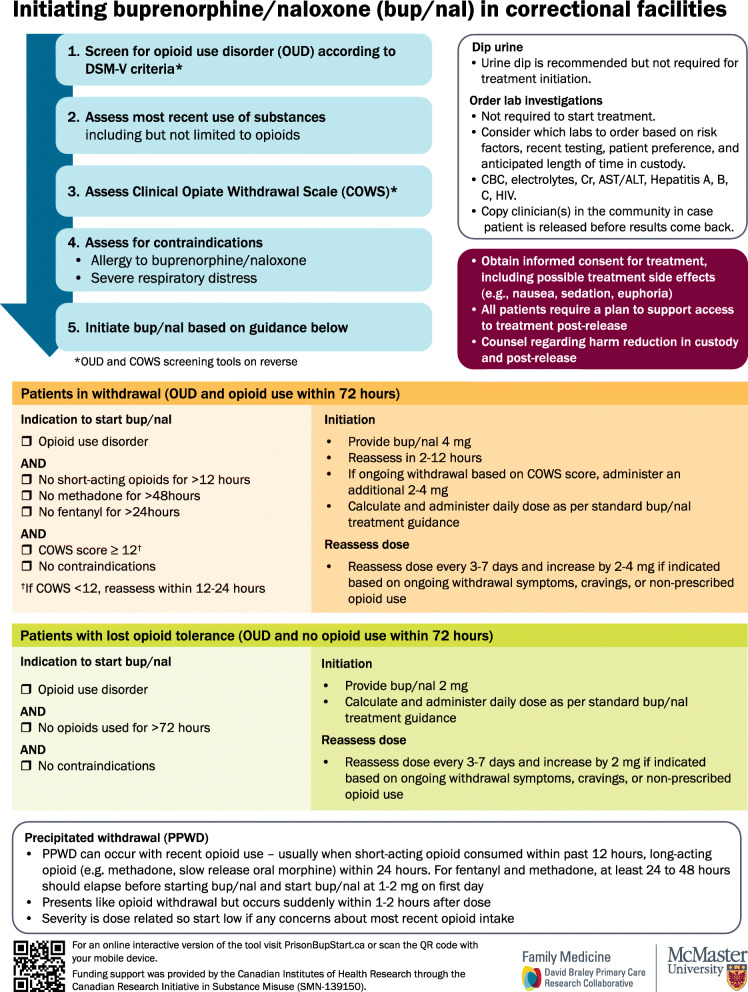
Tool to support evidence-informed buprenorphine/naloxone initiation in correctional facilities

We developed an engagement strategy to consult with relevant stakeholders, to ensure that the knowledge product would be tailored to the needs of our end users. Through our professional networks, we reached out to health care providers to discuss the utility of this tool to improve buprenophine/naloxone treatment access and quality, even in correctional facilities in which buprenorphine/naloxone was already available, and whether the steps of the tool would be feasible, useful, and scaleable in their correctional facilities. Specifically, we posted information on the tool in a national discussion forum for Family Physicians working in correctional facilities, and obtained feedback from one physician. We discussed the tool by phone and email with a nurse who had led the delivery of a correctional opioid agonist treatment program. We shared the tool by email with two physicians with addictions medicine expertise and obtained feedback. We planned to consult with people with lived experience of opioid use and incarceration regarding whether the tool was acceptable, for example, to explore what would be reasonable times to wait for treatment initiation after admission or for reassessment after initiation. We asked two clinicians if they could identify patients who would be willing to speak with us to provide feedback, invited our own patients to provide feedback, asked colleagues in non-governmental organizations if they could identify clients who would speak with us, and reached out to people in our networks with lived experience of incarceration. We spoke with only one person with lived experience of opioid use and incarceration. We engaged with fewer health care providers and people with lived experience than planned, which was likely due to difficulties related to accessibility and the limitations of telephone and video communications during the COVID-19 pandemic. We modified the tool iteratively based on these consultations. The current tool is shown in Fig. 2 and is publicly available online at PrisonBupStart.ca.

## Next steps

Following the development of this evidence-informed tool, we will continue to follow steps of the action cycle phase of the knowledge-to-action framework to implement this knowledge and tool into practice. This includes ongoing work with correctional authorities to support implementation and uptake by health care providers (*Select, tailor, implement interventions*). As immediate next steps, we will disseminate the tool and related knowledge translation materials to specific correctional health care providers and administrators through listservs and by mail, as well as promoting the tool through social media. We will deliberately engage with both health care providers and people with lived experience, and iteratively revise this tool as indicated, recognizing the limited engagement with these groups to date. As implementation proceeds, it will be important to monitor changes in provider knowledge (*Monitor knowledge use*), which was the target of this project, as well as impacts on outcomes (*Evaluate outcomes*) such as time to buprenorphine/naloxone initiation, the proportion of people with opioid use disorder requesting treatment who are initiated on it, overdose rates in correctional facilities and post-release, and ongoing substance use in custody and in the community.

Work is needed to address other barriers to treatment access for patients with opioid use disorder in correctional facilities as well as for patients with polysubstance use, and implementation and evaluation of this tool should proceed in parallel with efforts to address knowledge barriers. Further, a focus on harm reduction in correctional facilities and in the community is critical to support health for all people who use opioids, whether they have an opioid use disorder or not and whether they access treatment or not. Initiatives to improve treatment access and promote health should proceed in collaboration with people who experience incarceration and use drugs, as well as correctional authorities. Finally, attention is warranted to structures that affect health and health care for people who use drugs, including the criminalization of drug use and the governance of health care in correctional facilities.

## Data Availability

Not applicable.

## Abbreviations

COWS: Clinical Opiate Withdrawal Scale
OUD: Opioid Use Disorder
HIV: Human Immunodeficiency Virus
RCT: Randomized Controlled Trial

## References

[CR1] American Psychiatric Association. (2022). *What is a substance use disorder?* Retrieved February 16 from https://www.psychiatry.org/patients-families/addiction/what-is-addiction. Accessed 31 Jan 2022.

[CR2] Bruneau J, Ahamad K, Goyer ME, Poulin G, Selby P, Fischer B, Wild TC, Wood E (2018). Management of opioid use disorders: A national clinical practice guideline. CMAJ.

[CR3] Center for Substance Abuse Treatment (2004). Clinical guidelines for the use of buprenorphine in the treatment of opioid addiction. Treatment Improvement Protocol (TIP) Series, No. 40.

[CR4] Correctional Service of Canada. (2019). Opioid Agonist Treatment. Retrieved March 23, 2020 from https://www.csc-scc.gc.ca/health/002006-2007-en.shtml.

[CR5] Garcia CA, Correa GC, Viver AD, Kinlock TW, Gordon MS, Avila CA, Reyes IC, Schwartz RP (2007). Buprenorphine-naloxone treatment for pre-release opioid-dependent inmates in Puerto Rico. Journal of Addiction Medicine.

[CR6] Gordon MS, Kinlock TW, Schwartz RP, Fitzgerald TT, O'Grady KE, Vocci FJ (2014). A randomized controlled trial of prison-initiated buprenorphine: Prison outcomes and community treatment entry. Drug and Alcohol Dependence.

[CR7] Green TC, Clarke J, Brinkley-Rubinstein L, Marshall BDL, Alexander-Scott N, Boss R, Rich JD (2018). Postincarceration fatal overdoses after implementing medications for addiction treatment in a statewide correctional system. JAMA Psychiatry.

[CR8] Greenwald MK, Comer SD, Fiellin DA (2014). Buprenorphine maintenance and mu-opioid receptor availability in the treatment of opioid use disorder: Implications for clinical use and policy. Drug and Alcohol Dependence.

[CR9] Hedrich D, Alves P, Farrell M, Stover H, Moller L, Mayet S (2012). The effectiveness of opioid maintenance treatment in prison settings: A systematic review. Addiction.

[CR10] Kampman K, Jarvis M (2015). American Society of Addiction Medicine (ASAM) national practice guideline for the use of medications in the treatment of addiction involving opioid use. Journal of Addiction Medicine.

[CR11] Kouyoumdjian FG, Patel A, Kiefer L, Regenstreif L, To, M. J (2018). Physician prescribing of opioid agonist treatments in provincial correctional facilities in Ontario, Canada: A survey. PLoS One.

[CR12] Magura S, Lee JD, Hershberger J, Joseph H, Marsch L, Shropshire C, Rosenblum A (2009). Buprenorphine and methadone maintenance in jail and post-release: A randomized clinical trial. Drug and Alcohol Dependence.

[CR13] Marsden J, Stillwell G, Jones H, Cooper A, Eastwood B, Farrell M, Lowden T, Maddalena N, Metcalfe C, Shaw J, Hickman M (2017). Does exposure to opioid substitution treatment in prison reduce the risk of death after release? A national prospective observational study in England. Addiction.

[CR14] Nunn A, Zaller N, Dickman S, Trimbur C, Nijhawan A, Rich JD (2009). Methadone and buprenorphine prescribing and referral practices in US prison systems: Results from a nationwide survey. Drug and Alcohol Dependence.

[CR15] Ontario Ministry of the Solicitor General. (2019). *Verdict of the Coroner’s Jury, Office of the Chief Coroner, Inquest Chapman 2018*. Retrieved August 28 from https://www.mcscs.jus.gov.on.ca/english/Deathinvestigations/Inquests/Verdictsandrecommendations/OCCInquestChapman2018.html. Accessed 28 Aug 2021.

[CR16] Pearce, L. A., Min, J. E., Piske, M., Zhou, H., Homayra, F., Slaunwhite, A., … Nosyk, B. (2020). Opioid agonist treatment and risk of mortality during opioid overdose public health emergency: Population based retrospective cohort study. *BMJ, 368*. 10.1136/bmj.m772. 10.1136/bmj.m772 PMC7190018 32234712

[CR17] Springer SA, Chen S, Altice FL (2010). Improved HIV and substance abuse treatment outcomes for released HIV-infected prisoners: The impact of buprenorphine treatment. Journal of Urban Health.

[CR18] Straus, S., Tetroe, J., & Graham, I. D. (2013). *Knowledge translation in health care: Moving from evidence to practice*. Wiley. 10.1002/9781118413555. Accessed 28 Aug 2021.

[CR19] Sullivan LE, Moore BA, Chawarski MC, Pantalon MV, Barry D, O'Connor PG, Schottenfeld RS, Fiellin DA (2008). Buprenorphine/naloxone treatment in primary care is associated with decreased human immunodeficiency virus risk behaviors. Journal of Substance Abuse Treatment.

[CR20] Vocci FJ, Schwartz RP, Wilson ME, Gordon MS, Kinlock TW, Fitzgerald TT, O'Grady KE, Jaffe JH (2015). Buprenorphine dose induction in non-opioid-tolerant pre-release prisoners. Drug and Alcohol Dependence.

[CR21] Zaller N, McKenzie M, Friedmann PD, Green TC, McGowan S, Rich JD (2013). Initiation of buprenorphine during incarceration and retention in treatment upon release. Journal of Substance Abuse Treatment.

